# Prospective Application of Partially Digested Autologous Chondrocyte for Meniscus Tissue Engineering

**DOI:** 10.3390/pharmaceutics14030605

**Published:** 2022-03-10

**Authors:** Piya-on Numpaisal, Ching-Chuan Jiang, Chang-Hsun Hsieh, Hongsen Chiang, Chung-Liang Chien

**Affiliations:** 1 School of Orthopaedics, Institute of Medicine, Suranaree University of Technology, Nakhonratchasima 30000, Thailand; piya-on@sut.ac.th; 2 Department of Orthopaedic Surgery, National Taiwan University Hospital, Taipei 100, Taiwan; ccj@ntu.edu.tw (C.-C.J.); wwolf0220@gmail.com (C.-H.H.); 3 Department of Anatomy and Cell Biology, College of Medicine, National Taiwan University, Taipei 100, Taiwan

**Keywords:** meniscus cell, sustainable tissue engineering, cell-based therapy, chondrogenic expression, chondrogenic property, cell proliferation

## Abstract

Background: Meniscus tissue engineering has yet to achieve clinical application because it requires chondrogenic induction and in vitro cell expansion. Contrarily, cartilage engineering from autologous chondrocytes has been successfully applied in one-stage surgery. If the natural chondrogenic potential of meniscus cells can be demonstrated, meniscus tissue engineering would have more value in clinical settings. Materials and Methods: In total, 10 menisci and pieces of cartilage were obtained during total knee replacements. The tissues were collected for cell isolation and expansion. Their chondrogenic properties were examined by immunohistofluorescence and gene expression analyses. Results: In native cartilage, immunofluorescence demonstrated the presence of collagen I, aggrecan, and traces of collagen I, whereas comparable staining was seen in the inner and middle meniscus. The presence of collagen I but the absence of collagen II and aggrecan were observed in the outer meniscus. In passage 2, chondrocytes showed the presence of collagen II and aggrecan, and the absence of vimentin. The vimentin and aggrecan staining were comparable in the inner and middle meniscus cells, whereas the outer cells showed only vimentin staining. In the gene expression analyses, the expressions of collagen II and aggrecan in the native chondrocyte and the inner and middle meniscus were higher than those of the cells from the outer meniscus, but they were not different in collagen I. In the passage 2 culture, chondrocytes had a higher expression of collagen II and aggrecan than the meniscus cells. Cells from the inner and middle areas had higher collagen II and aggrecan expression than those from the outer meniscus. Conclusion: Without chondrogenic induction, inner and middle meniscus cells possess a chondrogenic phenotype. Specifically, native meniscus cells exhibited more robust chondrogenic potential compared with those of the passage 2 monolayer culture.

## 1. Introduction

The fibrocartilagenous meniscus of the knee functions to protect articular cartilage for both load transmission and shock absorption across the joint [1,2]. For this reason, the meniscus is vulnerable to injuries. Meniscus injury can occur in any area: the avascular inner zone, the transitional middle zone, and the vascular outer zone. Specifically, inner meniscus injury is regarded as the most challenging for orthopaedic surgeons, due to its poor healing potential. After surgical repair, only 25% of extensive tears at this part would heal 18 months after the operation [3]. Moreover, symptomatic inner meniscal tear is commonly treated with partial meniscectomy by excising unhealed meniscal fragments; however, such a procedure can contribute to the subsequent degeneration of the knee joint [4].

It is speculated that a lack of blood supply is the cause of the poor healing of the inner meniscus, as the peripheral meniscus is vascularized while the inner meniscus is not [5]. Such speculation was supported by the finding that more than 80% of the patients receiving meniscal repair with the concomitant reconstruction of the anterior cruciate ligament had an asymptomatic tibiofemoral joint and did not need subsequent surgery [6]. Nevertheless, various strategies providing supplemental blood or blood components have been proposed to promote meniscal healing after surgical repair, but the results are inconclusive [7,8]. To overcome this problem, many studies were conducted in order to provide a greater understanding of meniscus tissue, and found that the inner meniscus possesses chondrogenic properties, similar to hyaline cartilage. Verdonk et al. demonstrated that most meniscus cells in alginate gel had high collagen I and aggrecan production [9]. By focusing on specific areas of the meniscus, some studies have shown that—under chondrogenic induction by growth factors—meniscus cells from the inner part had a higher chondrogenic phenotype than those from the outer part [10]. This knowledge has been employed in meniscus tissue engineering, and has shown promising results [11,12]. Scotti and colleagues reported better neotissue formation by using chondrocyte-seeded fibrin glue to fill meniscus gaps [11]. In the same way, Kobayashi et al. reported extracellular matrix enhancement in rabbit meniscus defect after implanting meniscus fragments and wrapping them with fascia. In addition, chondrocyte-like cells in the defects were seen upon histological examination [13]. More recently, Shimomura et al. demonstrated the better healing of radial meniscus tears in bovine explant models using polycaprolactone-incorporated polyethylene oxide scaffold seeded with meniscus fibrochondrocytes wrapping around the tear sites [14]. Although the impressive results of meniscus tissue engineering have been proven, meniscus tissue engineering has yet to achieve clinical application, as it still requires ex-vivo cell expansion, chondrogenic induction by growth factors, and a two-step surgical procedure.

Compared to avascular hyaline cartilage, it was once believed that articular cartilage, “once destroyed, is not repaired” [15], as said by Dr. William Hunter in 1743. However, as more evidence points to the causal relationship of articular cartilage damage and the early degeneration of the knee, rigorous investigations have been underway over the past decades to understand the chondrogenic nature of chondrocytes in the hyaline cartilage, cartilage repair and regeneration [16,17]. The knowledge from these studies has then been applied in cartilage tissue engineering. Currently, autologous chondrocyte implantation (ACI), which is claimed to create hyaline cartilage-like tissue, is approved by the US Food and Drug Administration for use as a treatment option for focal cartilage defects. ACI is a cell-based cartilage repair that consists of two stages of surgery: (1) an ex-vivo expansion of autologous chondrocytes, and (2) the implantation of the expanded cells into the cartilage defect. However, ACI treatment is not without drawbacks: a major issue with ACI is the loss of the chondrogenic phenotype from in vitro expansion [18]. Moreover, because ACI is a two-stage procedure, it is not the preference for some orthopaedic surgeons. In order to eliminate these problems, other approaches for cell-based therapy have been investigated. Beakers et al. used chondrocytes with the remaining extracellular matrix called chondrons, combined with mesenchymal stem cells (MSCs) and implanted into a cartilage defect of goats [19]. The result showed the improvement of cartilage regeneration in microscopic, macroscopic, and biochemical data. A clinical trial by Chiang et al. demonstrated a lower pain score at 3 months and improvement of the Knee injury and Osteoarthritis Outcomes Score (KOOS) at a two-year follow-up [20] using rapidly digested cartilage pieces combined with a biodegradable biphasic scaffold to treat focal cartilage defects. According to recent studies, it is very interesting that fewer biological processes can offer the promising results of cell-based therapy. The key advantages of this technique are the use of autologous tissue without additional growth factors, and that it can be completed in one surgical setting.

Taken together, the potential solution directs us to sustainable tissue engineering, which is more applicable for clinical practice. The key success of this approach is to prove that freshly harvested cells from the native meniscus and cartilage have fundamental chondrogenic potential in a chondrogenic induction-free environment. If this is proven, a single-step autologous cells implantation would be a fascinating treatment option for avascular-area meniscus repair. Therefore, our study aimed to compare the chondrogenic phenotypes of the cells from the native meniscus and culture-expanded cells by using chondrocytes from hyaline cartilage as a reference.

## 2. Materials and Methods

### 2.1. Sample Collection and Preparation

The study was conducted after approval by the Institutional Review Board for Research Ethics in the corresponding author’s hospital, where all the patients were recruited and underwent surgeries. We recruited patients younger than 70 years old, who were scheduled to receive prosthetic total knee replacements for primary osteoarthritis. Patients with other types of inflammatory arthropathy, e.g., rheumatoid and gouty arthritis, or arthritis secondary to previous injuries, such as the rupture of cruciate ligaments or intra-articular fractures, were excluded. Every enrolled patient signed an informed consent form to participate in the study.

**Figure 1:**
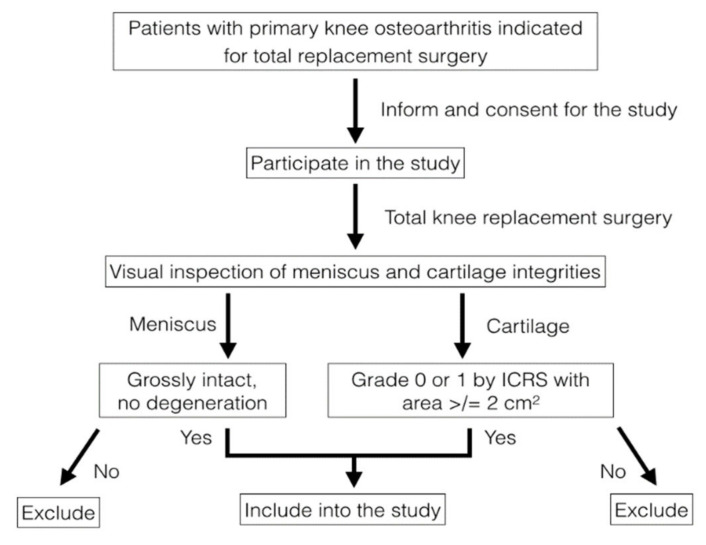
Flowchart of the sample collection. The menisci and femoral condyles of the patients who met the criteria and volunteered to join the study were visually inspected; only patients who had non-degenerative meniscus and spared at least 2 cm^2^ of grade 0 and 1 cartilage by ICRS grading were included in the study.

Specimens were successfully collected from 10 patients: all were females, and their mean age was 68.7 years. The specimens were processed as shown in Figure 1. During surgery, the integrity of the lateral menisci was visually inspected. The menisci, absent significant defects or degeneration from gross appearance, were excised carefully along their peripheral attachments with the synovial tissue, and were harvested as a whole piece. Whole pieces of femoral condyles were concomitantly collected. The tissue samples were washed with normal saline to remove synovial fluid and debris. The tissues were then kept at 4 °C in normal saline and processed within 24 h.

**Figure 2:**
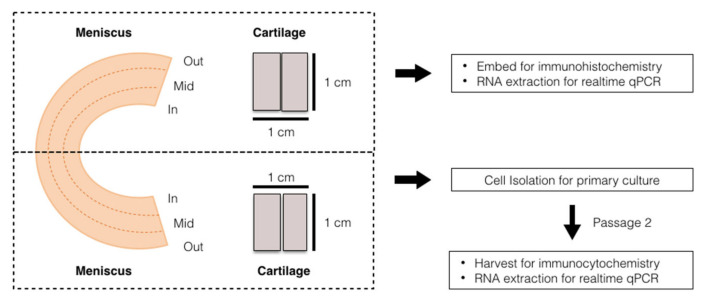
Diagram of the sample preparation. The prepared meniscus and cartilage tissues from individual patients were equally separated into two portions: the first portion was used for histology and native cell RNA extraction, and the other portion was used for cell isolation, then cultured into passage 2 before we proceeded with the other analyses.

Immediately prior to the histological study and cell isolation, the samples were prepared as shown in Figure 2. All of the synovial tissues were removed and washed with phosphate-buffered saline (PBS, Sigma-Aldrich, St. Louis, MO, USA). The femoral condyles were washed and examined in order to identify areas that presented as grade 0 or 1 according to the International Cartilage Repair Society grading [21]. From the superficial through to just above the calcified zone, the cartilage was harvested using a scalpel; cartilage pieces were gathered to obtain 2 cm^2^ in total. Individual menisci were cut radially at mid-body; one half was used for immunohistochemistry and ribonucleic acid (RNA) extraction, and the other half proceeded to cell isolation and then primary culture. In the same way, the cartilage pieces were equally divided, 1 cm^2^ for native tissue analysis and 1 cm^2^ for the cell culture.

### 2.2. Histology and Immunofluorescent Analysis

The menisci were cut radially at their body to obtain a 5 mm width for histological study. The samples for histological study were processed as previously described [10]. In brief, the samples were fixed in 10% neutral buffered formalin at 4 °C for 4–6 h, cryoprotected with 30% sucrose at 4 °C overnight, then transferred to cryomolds with frozen sectioned compound embedding medium (Lieca Biosystems, Buffalo Grove, IL, USA) and rapidly frozen in liquid nitrogen-cooled isopentane. The frozen tissue blocks were sectioned with a cryostat for microslides, which were stored at −80 °C.

Before staining, the slides were dried at room temperature for 30 min, fixed with acetone at −20 °C for 10 min, then allowed to dry for 5 min, washed with PBS, and treated with 0.1% Triton X-100 in PBS for 15 min at room temperature. The antigens were retrieved with 0.5% trypsin for 10 min at 37 ºC, and were blocked for 1 h at room temperature in PBS supplemented with the mixture of 1% bovine serum albumin (BSA), 0.1% gelatin, 0.05% Tween-20, and 2% normal goat serum. The sections were incubated overnight at 4 °C with primary antibodies in solutions of PBS plus 1% BSA and 0.1% gelatin. The primary antibodies (Table 1) were used in the order of: mouse monoclonal anti-type I collagen at 1:200, rabbit polyclonal anti-type II collagen at 1:100, and mouse monoclonal anti-aggrecan at 1:100.

The specimens of articular cartilage were trimmed, embedded with frozen sectioned compound embedding medium, frozen in liquid nitrogen-cooled isopentane, and sectioned with a cryostat for the microslides. The slides were fixed in acetone for 10 min at −20 °C, washed and treated for antigen retrieval as previously described, and blocked with 10% fetal bovine serum (FBS, Gibco, Life Technologies, Grand Island, NY, USA) for 30 min at room temperature. The primary antibodies were then applied, as for the treatment of meniscus samples.

Both the specimens of meniscus and articular cartilage were treated with secondary antibodies of goat anti-rabbit IgG-FITC and goat anti-mouse IgG-TRITC at 1:200 dilutions for 1 h at room temperature. The cellular nuclei were stained with Hoechst 33,342 (Thermo Scientific, Rockford, IL, USA) at 1:1000 dilution. The negative controls were prepared by omitting the primary antibodies from the protocol. Images were acquired with a confocal microscope (Leica TCS SP5, Leica, Heidelberg, Germany). The contrast and brightness of the photomicrographs were adjusted with Adobe Photoshop CS2 software (Adobe Systems Software Ireland, Ltd., Dublin, Ireland).

### 2.3. Cell Isolation, Culture and Immunocytofluorescent Analysis

Half of an individual meniscus was trisected along the radial width into inner, middle, and outer thirds. Each zone of meniscus tissue was separately trimmed and gathered to obtain a 1 cm^3^/zone; furthermore, pieces of articular cartilage of similar volume were prepared for subsequent assay. These samples were chopped and digested with 0.1% collagenase (Worthington Biochemical, Lakewood, NJ, USA) overnight at 37 °C to free the cells. The cells were centrifuge-collected at 1500 rpm for 5 min, washed 3 times with PBS, and cultured in a monolayer with high-glucose Dulbecco’s modified Eagle’s medium (DMEM/F12, Gibco, Life Technologies, Carlsbad, CA, USA) supplemented with 10% FBS and an antibiotic–antifungal mixture in a humidified, 37 °C, 5% carbon dioxide environment. Some cells were collected immediately following the collagenase digestion, and were analyzed as native cells before expansion. The others were plated and cultured, with the medium being refreshed every 3 days, and were passaged when they reached 95% confluence. The passage 2 cells were used for further studies.

Approximately 1 × 10^4^ cells from each area of meniscus and cartilage were used for the immunocytofluorescent analysis after being incubated separately in 12-well plates at 37 °C for 48 h. The cells were collected, washed with PBS, fixed with cold methanol in −20 °C for 10 min, blocked with 10% FBS in PBS for 30 min at room temperature, and incubated with primary antibodies at 4 °C overnight. The primary antibodies included mouse monoclonal anti-vimentin at 1:100, anti-type I collagen at 1:200, and rabbit polyclonal anti-type II collagen and mouse monoclonal anti-aggrecan at 1:100 dilutions (Table 1). The specimens were then treated with secondary antibodies containing goat anti-rabbit IgG-FITC and goat anti-mouse IgG-TRITC at 1:200 dilutions. The cellular nuclei were stained with Hoechst 33,342 at 1:1000 dilutions. The negative controls were prepared, and the images were acquired and adjusted as described above.

### 2.4. Quantitative Analysis for Gene Expression

The total RNA was extracted from the cells to be subjected to quantitative gene-analysis, either for native or passage-2 cells, with an RNeasy mini Kit (Qiagen, Hilden, Germany). The quantitative real-time polymer chain reaction (qRT-PCR) was performed with a SensiFAST SYBR and Fluorescein Kit (BIOLINE, London, UK) on the target genes, including collagen I, collagen II and aggrecan, with 18S rRNA serving as the housekeeping gene (Table 2).

### 2.5. Statistical Analysis

The statistical analysis was carried out using SPSS software (SPSS version 20, IBM, Armonk, NY, USA). Due to the fact that the Kolmogorov–Sminov test showed non-normally distributed data, the Krukal–Wallis H test was used with Dunn’s post hoc testing instead of ANOVA. A *p*-value of <0.05 was considered statistically significant.

## 3. Results

### 3.1. Tissues and Native Cells

**Figure 3:**
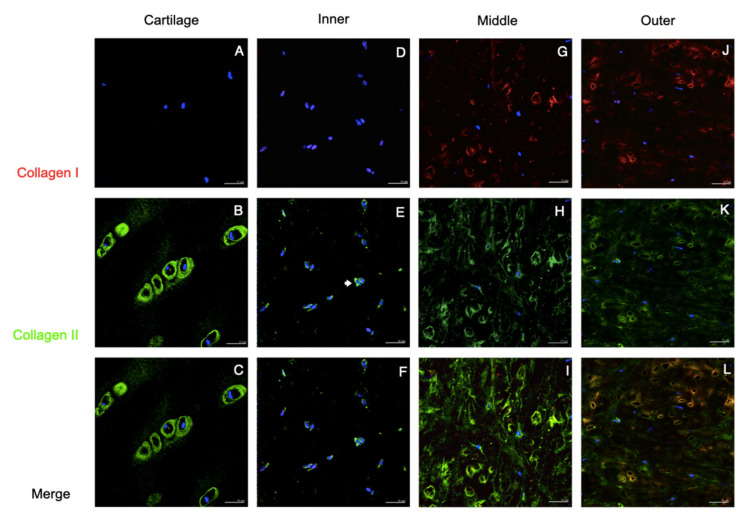
Collagen I and collagen II staining of the native tissues. Immunofluorescent localized type I and II collagen in human cartilage and meniscus. Positive staining of Col II in the pericellular matrix was observed in the cartilage, which was called chondron (**B**,**C**), and was scantly demonstrated in the meniscus (arrows in (**E**,**H**,**K**)). Collagen I staining presented in middle and outer meniscus (**G**,**J** and **K**) but absented in cartilage and inner meniscus (**A**,**D**). The co-localization of Col I and Col II were displayed in the middle and outer meniscus (arrow heads in **I** and **L**). In the merged channel, the inner and middle meniscus presented toward chondrogenic staining (**F**,**I**), whereas the outer meniscus showed more fibrogenic-like staining (**L**). Scale bars: 25 µm.

**Figure 4:**
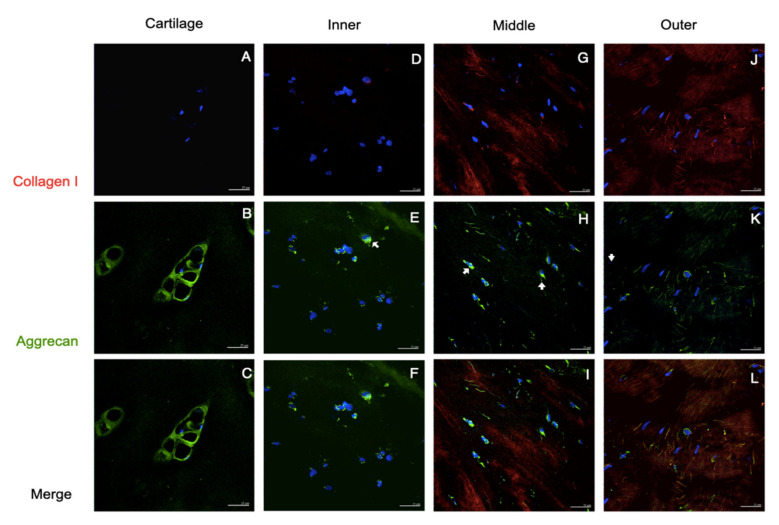
Collagen I and aggrecan staining of native tissues. Immunofluorescent localized Col I and Agg in human cartilage and meniscus. No positive staining of Col I was seen in the cartilage (**A**), but a tiny stain was exhibited in the inner meniscus (arrowhead in (**D**)); apparently, Col I staining was detected in the middle and outer parts (**G**,**J**). The Agg staining obviously presented the pericellular matrix of the cartilage (**B**,**C**), and was scarcely displayed in three areas of the meniscus (arrows in (**E**,**F**,**H**,**I**,**K**,**L**)). Scale bars: 25 µm.

The immunostaining of collagen I and II was demonstrated in Figure 3. The staining of cartilage tissue showed an absence of collagen I but an abundance of collagen II, which accumulated primarily in the pericellular matrix region. The inner meniscus also had similar staining patterns but less intense than those in the cartilage. In the staining of the middle and outer meniscus, the majority of collagen I and collagen II had fibrillar-like patterns. In the merge channel, the co-localization of collagen I and II was observed in the middle and outer meniscus, but the middle meniscus had predominant collagen II staining whereas the outer meniscus presented more staining of collagen type I than collagen type II. In Figure 4, the staining of collagen I and aggrecan is shown. Again, the cartilage had aggrecan staining accumulated in the pericellular matrix, whereas the staining of collagen I was absent. In the inner meniscus, the pericellular staining of aggrecan was also observed, but was not as obvious as that in the cartilage, and the staining of collagen I was scanty. In the middle and outer meniscus, collagen I staining was present in most areas, while aggrecan staining was seen around the cells.

### 3.2. The Passage 2 Cultured Cells

**Figure 5:**
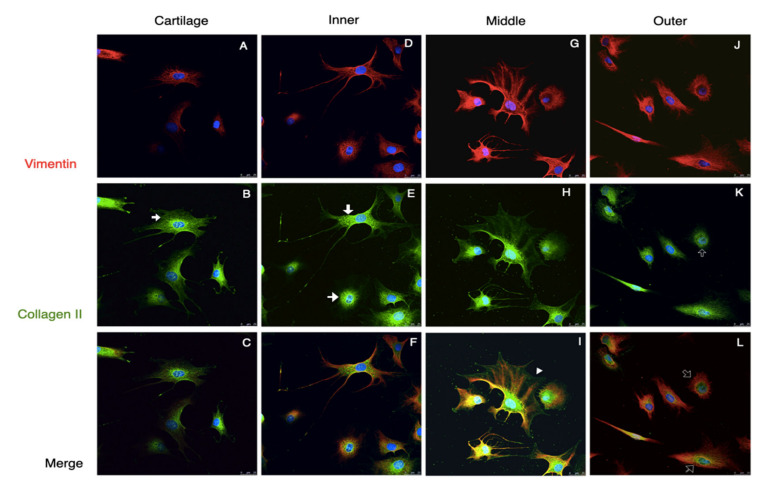
Collagen II and vimentin staining of the passage 2 cultured cells. Immunofluorescence demonstrated the vimentin and Col II staining of passage 2 cultured cells from the cartilage and meniscus. Two distinct cell morphologies were demonstrated in the meniscus. Vimentin staining presented a cytoskeletal-like pattern (**A**,**D**,**G**,**J**). Col II in the cartilage and inner cells exhibited fine mesh-like patterns, particularly in the cytosol (arrows in (**B**,**E**)), but cells from the middle meniscus had overspread Col II from the cytoplasm along to the cells’ process (arrowheads in (**H**)) whereas Col II in the outer meniscus cells was limited in the perinuclei area (hollow arrows in (**K**)). In the merged channel, inner and middle meniscus presented both chondrogenic and fibrogenic staining (**F**,**I**), whereas the outer meniscus cells showed more fibrogenic-like staining (**L**) and chondrocyte demonstrated chondrogenic staning (**C**). Scale bars: 25 µm.

**Figure 6:**
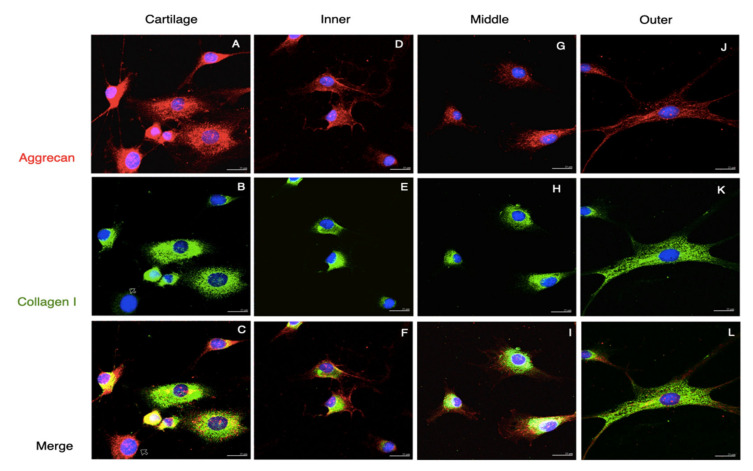
Collagen I and aggrecan staining of passage 2 cultured cells. Immunofluorescence demonstrated the Agg and Col I staining of passage 2 cultured cells from the cartilage and meniscus. Agg staining displayed punctuate patterns scattered in the cells (arrow in (**A**,**C**,**D**,**F**,**G**,**I**,**J**,**L**)), whereas Col I staining was mostly found around the nuclei (arrowheads in (**B**,**E**,**H**)). Some cells from the cartilage were absent of Col I staining (hollow arrows in (**B**)). And cells from outer meniscus clearly presented Col I staining (**K**,**L**). Scale bars: 25 µm.

Generally, there were two distinct morphologies of passage 2 cultured meniscus cells: the oval–polygonal cell with a big, round nucleus, and the fusi-form cell with a small, elliptical nucleus, long processes and less cytoplasm (Figure 5). Most of the outer meniscal cells had smaller nuclei and scanty cytoplasm compared with the chondrocyte or those cells from the inner and middle meniscus. The chondrocytes had strong staining of collagen II and weak staining of vimentin. The cultured meniscal cells, regardless of their shape, contained both collagen II and vimentin, but varied in distribution. The positive staining of collagen II around the nuclei was detected in the inner and middle meniscus cells, whereas vimentin appeared primarily along the cell processes (Figure 5F,I). Collagen II in the perinuclei area was detected in the outer meniscus cultured cells but appeared less intense than vimentin (Figure 5L). All of the groups of passaged II cultured cells had positive staining of both aggrecan and collagen I; the aggrecan staining showed scattered, punctuated patterns all over the cytoplasm, whereas collagen I existed primarily in the perinuclei (Figure 6, red and green channels). The absence of collagen I staining in some cartilage cells was observed (Figure 6B, hallow arrow). In the comparison of the merged images, faintly stained aggrecan in outer meniscus cells was noted (Figure 6).

### 3.3. Gene Expression Analyses

**Figure 7:**
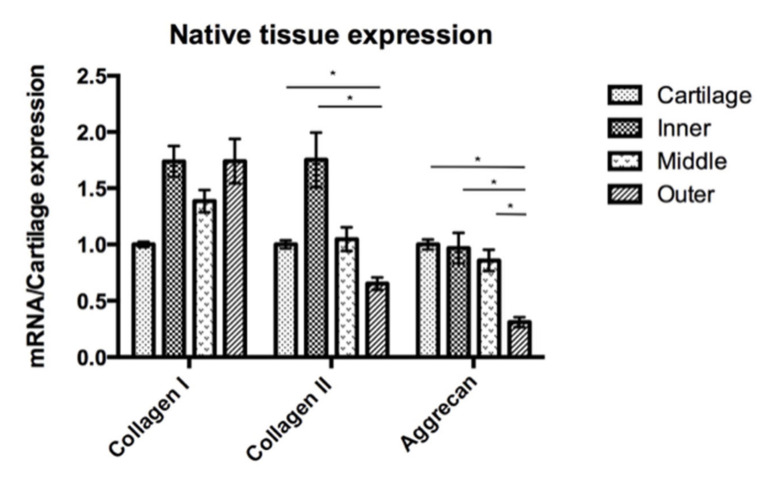
Native cartilage and meniscus tissue expression with the COL1A1, COL2A1 and ACAN gene expression of the meniscus is presented in comparison to the cartilage gene expression. The expression of COL2A1 was higher in the cartilage and inner meniscus compared to the outer meniscus, but there were no significant differences between the cartilage and the inner and middle meniscus. For ACAN, the outer meniscus had the lowest expression, and there were no significant differences among the other three groups. *: *p*-value < 0.05.

**Figure 8:**
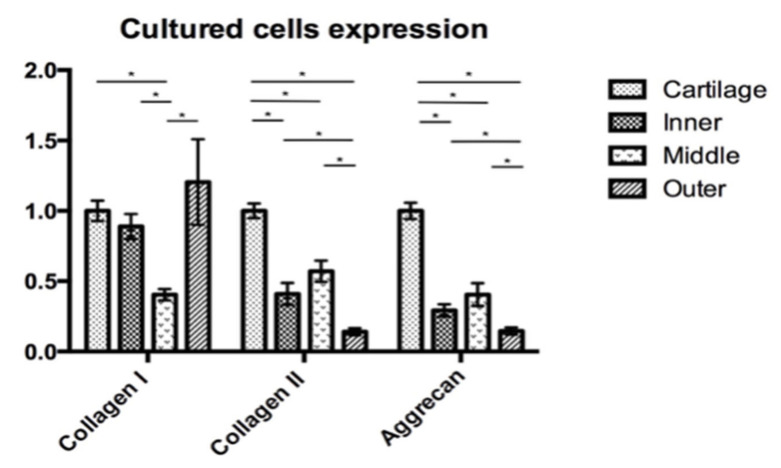
Cartilage and meniscus primary cultured cell expression with the COL1A1, COL2A1 and ACAN gene expression of passage 2 meniscus cultured cells is presented in comparison with the passage 2 chondrocyte culture. The middle meniscus cells had the lowest COL1A1 expression, whereas the expression in the other three groups was not significantly different. The COL2A2 and ACAN expressions were lower in the meniscus cells compared to the chondrocyte. Among the meniscus cells, the inner and middle meniscus had higher COL2A2 and ACAN expressions compared with those from the outer meniscus. The inner and middle meniscus had no significant differences of gene expression. *: *p*-value < 0.05.

In order to compare the target gene expression, the expression of articular chondrocytes was used as a reference. In the native cells, qRT-PCR showed a different expression of COL1A1, COL2A1 and ACAN by the native chondrocytes and meniscus cells (Figure 7). The expressions of collagen I of the four different cell groups were comparable. The expression of COL2A1 was significantly lower in the outer meniscus cells (0.65) compared with those in chondrocytes (1, *p* = 0.025) or inner meniscus cells (1.75, *p* = 0.046). The expression of COL1A2 by middle meniscus cells (1.05) was not significantly different from those of the inner or outer meniscus cells. The expression of ACAN by the chondrocytes was comparable with the expressions of the inner and middle meniscus cells (inner = 0.97, middle = 0.86). The outer meniscus cells had a significantly lower expression ACAN (0.31) than the chondrocytes (1, *p* < 0.001), and the inner (0.97, *p* = 0.030) and middle meniscus cells (0.86, *p* = 0.031), while the latter two had a comparable expression. The qRT-PCR analyses of passage 2 cultured cells showed the expressions of the target genes in Figure 8. Regarding the expression of COL1A1, there was no significant difference between the chondrocytes and the inner or outer meniscus cells, while the cells from the middle meniscus had a lower COL1A1 expression than the chondrocytes (chondrocyte = 1, middle = 0.40, *p* < 0.001), inner meniscal cells (0.89, *p* = 0.001) and outer meniscus cells (1.20, *p* = 0.047). The chondrocytes had a higher expression of COL2A1 compared with the cells from the meniscus (inner = 0.41, *p* < 0.0001, middle = 0.57, *p* < 0.001, outer = 0.14, *p* < 0.0001), which was the same as the expression of ACAN (inner = 0.29, *p* < 0.0001, middle = 0.40, *p* < 0.0001, outer = 0.15, *p* < 0.0001). Among the three groups of meniscus cells, cells from the inner and middle meniscus had a comparable expression of COL2A1 and ACAN, but cells from the outer meniscus had a lower expression (COL2A1; inner; *p* = 0.023, middle; *p* < 0.001 and ACAN; inner *p* = 0.028, middle *p* = 0.039).

## 4. Discussion

The function of the tissue is determined by its organization and composition. In order to withstand a compressive load, the articular cartilage predominantly relies on collagen II and aggrecan as fundamental components of the extracellular matrix [22]. In the meniscus, the avascular inner zone primarily bears the compression, whereas the outer meniscus withstands tensile force from hoop stress. Therefore, the extracellular matrix of the meniscus differs between the areas. In our study, the immunofluorescence of the native meniscus resembled that of previous studies [22,23]: the inner meniscus carried more collagen type II and aggrecan than collagen type I; the middle transitional zone showed a comparable expression of collagen type II, aggrecan, and collagen type I; the outer meniscus showed predominantly the presence of collagen type I. Based on the immunofluorescent results, we believe that such an organization of the matrix corresponds with the meniscus function. This is because aggrecan is a large proteoglycan and possesses a highly negative charge, such that it interacts intimately with the polar molecules of water and makes the matrix a hydrophilic sponge [24]. Additionally, together with the collagen II fibers that constitute a supporting mesh to contain the aggrecan molecules, this integration provides the inner meniscus with compressive resistance [25]. On the other hand, the greater amount of collagen I fibers are organized as a dense complex network in the outer meniscus in order to withstand hoop stress [26]. Such local variations of tissue composition were compatible with the immunocytochemical findings on regional cells. The passage 2 cultured cells from the three areas of the meniscus showed combined chondrogenic and fibrogenic expression, but the chondrogenicity was predominant in the inner and middle meniscus cells, whereas the fibrogenic phenotype exceeded in cells from the outer meniscus.

Although recent studies have reported the chondrogenic phenotype of inner meniscus cells, the results were based on exogenous chondrogenic stimuli such as growth factor supplements, mechanical stimulation, and 3-dimensional culture [27,28]. In our study, the meniscus cells were cultured in a simple expanded medium, and we found that their phenotypes varied between different regions of the meniscus. The inner and middle meniscus cells had more chondrogenic potential, whereas the outer meniscus cells expressed more fibrogenicity. This is is compatible with the findings of a previous study [29], which showed that cells from the inner meniscus had greater potential toward chondrogenesis, while outer meniscal cells had a wider spectrum of phenotypes, undergoing chondrogenic, adipogenic and osteogenic differentiation.

Furthermore, in our study, native cells from the inner and middle meniscus expressed higher COL2A1 and ACAN, which hinted at differentiation towards a chondrogenic phenotype. However, this finding could not be observed in passage 2 cultured cells. It was pointed out that time and passaging decreased the chondrogenic properties of meniscus cells, which is similar to the chondrocytes of articular cartilage [30]. The chondrocyte dedifferentiation could be explained by the previous studies by Gailuk, who found that chondrocytes were surrounded by pericellular matrix, and that the signal from either biochemical or biomechanical interaction from the extracellular matrix to the chondrocytes was determined by the surrounding pericellular matrix [31]. The soluble biochemical agents could be changed or retained after passaging [32]. Therefore, the passaged cells may lose their cellular properties after expansion. The importance of the pericellular matrix of the chondrocytes was also demonstrated by Larson and his colleague. They compared pallet cultures of isolated chondrocytes and the remaining pericellular matrix chondrocytes or chondrons at 8 weeks after treatment. The chondron-pallet group had more collagen II deposition in the extracellular matrix and better proteoglycan production compared to the chondrocyte pallets [33]. Moreover, Bekkers reported a better cartilage-specific matrix production outcome of the co-cultured combination of chondron and MSCs than the combination of chondrocytes and MSCs [19]. Therefore, the better chondrogenic phenotypes of native meniscus cells in our study could be from the effect of the remaining pericellular matrix.

**Figure 9:**
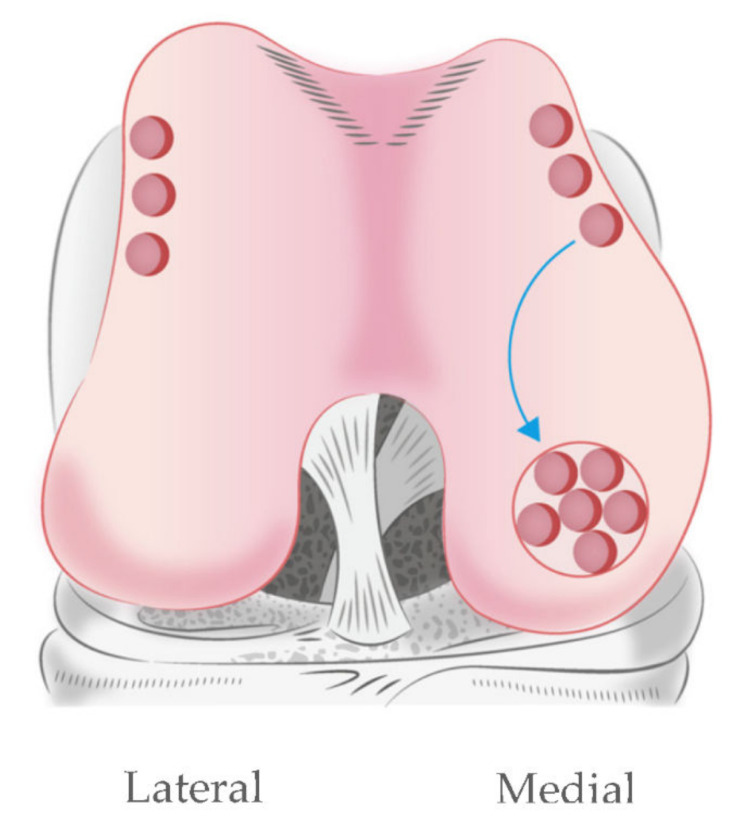
Mosaicplasty at the medial femoral condyle using autologous osteochondral plugs. The donor site is a non-weight-bearing area of distal femur at the medial and lateral edges of the femoral condyle.

Regarding clinical application, the harvesting of meniscus tissue for use as a cell source is hardly possible. The meniscus is firmly attached to the surrounding structure in the knee joint, and any resection of the meniscus would disrupt the collagen fibers and deduct meniscus functions [4]. In the knee joint, the lateral edge of the anterior femoral condyle is a non-weight-bearing area (Figure 9); it has been used as a donor site for the osteochondral plug of mosaicplasty or osteochondral autograft transfer (OAT). This treatment option has been employed for cartilage injury treatment for several years, and is reported as having minimal donor site complications in terms of contact pressure [34,35]. Our results demonstrated that hyaline cartilage and the inner meniscus possess similar components [36]. Thus, hyaline cartilage from lateral femoral condyle would be a good candidate for partially digested chondrocyte for cell-based therapy meniscus. According to the literature review, osteochondral plugs can be applied for 3.5 cm^2^ cartilage lesions, which is adequate to replace the meniscus defect.

## 5. Conclusions

In conclusion, our study supported the supposition that different areas of meniscus presented different cellular components and extracellular matrices according to their functions. The inner meniscus and hyaline cartilage function to withstand compressive load, and presented more chondrogenic properties in a non-chondrogenic induction environment. Moreover, the remaining pericellular matrix meniscus cells and chondrocytes had more robust chondrogenic phenotypes compared to the passage 2 cultured cells. For sustainable application, freshly isolated chondrocytes from non-weight-bearing femoral condyles could be used as a cell source in single-step cell-based therapy meniscus tissue engineering.

## Figures and Tables

**Table 1:** List of antibodies applied for the immunochemistry.

**Antigen**	Antiserum	Cell Type	Titer
Aggrecan	$Mousemonoclonalanti-aggrecan^{\;1}$	Chondrocyte	1:100
Collagen I	$Mousemonoclonalanti-aggrecan{\;}^{2}$	Fibroblast	1:200
Collagen II	$Rabbitpolyclonalanti-collagenII^{\;3}$	Chondrocyte	1:100
Vimentin	$Mousemonoclonalanti-vimentin^{\;4}$	Fibroblast	1:100
Mouse IgG	$Goatanti-mouseIgGFITCandTRITC^{\;5}$		1:200
Rabbit IgG	$Goatanti-rabbitIgGFITC{\;}^{6}$		1:200

^1^ Abcam, Cambridge, UK (Cat. No. AB3778); ^2^ Abcam, Cambridge, UK (Cat. No. AB90395); ^3^ GeneTex, Irvine, CA, USA (Cat. No. GTX20300); ^4^ Santa Cruz Biotechnology, Dallas, TX, USA (Cat. No. sc-80975); ^5^ Sigma-Aldrich, Saint Louis, MO, USA (Cat. No. F9887/T5393); ^6^ Sigma-Aldrich, Saint Louis, MO, USA (Cat. No. F0382).

**Table 2:** Sequences of each primer set for qRT-PCR.

Genes	Pairs	Primer Sequence (5′ to 3′)
Type I collagen (COL1A1)	Sense Antisense	GGAGGAGAGTCAGGAAGG GCAACACAGTTACACAAGG
Type II collagen (COL2A1)	Sense Antisense	GGCAGAGGTATAATGATAAG ATGTCGTCGCAGAGG
Aggrecan (ACAN)	Sense Antisense	ATACCGTCGTAGTTCC TCCTTGTCTCCATAGC
18S rRNA	Sense Antisense	ATACCGTCGTAGTTCC GTCTCGTTCGTTATCG

## Data Availability

The datasets were used and/or analyzed during the current paper.

## Abbreviations

ACI	Autologous Chondrocyte Implantation
KOOS	Knee injury and Osteoarthritis Outcomes Score
PBS	Phosphate-buffered Saline
RNA	Ribonucleic Acid
BSA	Bovine Serum Albumin
FBS	Fetal Bovine Serum
COL1A1	Collagen type 1 alpha 1 gene
COL2A1	Collagen type 2 alpha 1 gene
ACAN	Aggrecan gene
18S rRNA	18S ribosomal RNA
MSCs	Mesenchymal Stem Cells
